# Stress-Induced Immunosuppression Affects Immune Response to Newcastle Disease Virus Vaccine via Circulating miRNAs

**DOI:** 10.3390/ani12182376

**Published:** 2022-09-12

**Authors:** Yufei Tian, Yang Liu, Qiuyuan Wang, Jie Wen, Yiru Wu, Jianwei Han, Chaolai Man

**Affiliations:** College of Life Science and Technology, Harbin Normal University, Harbin 150001, China

**Keywords:** chicken, circulating miRNA, stress-induced immunosuppression, Newcastle disease, vaccine

## Abstract

**Simple Summary:**

Circulating miRNAs play important roles in immune response and stress-induced immunosuppression, but the function and mechanism of stress-induced immunosuppression affecting the NDV vaccine immune response remain unknown. In our study, key timepoints, functions, mechanisms, and potential biomarkers of circulating miRNAs involved in immune response and immunosuppression were discovered, providing a theoretical basis for studying the roles of circulating miRNAs in immune regulation.

**Abstract:**

Studies have shown that circulating microRNAs (miRNAs) are important players in the immune response and stress-induced immunosuppression. However, the function and mechanism of stress-induced immunosuppression affecting the immune response to the Newcastle disease virus (NDV) vaccine remain largely unknown. This study analyzed the changes of 15 NDV-related circulating miRNAs at different immune stages by qRT-PCR, aiming to explore the key timepoints, potential biomarkers, and mechanisms for the functional regulation of candidate circulating miRNAs under immunosuppressed conditions. The results showed that stress-induced immunosuppression induced differential expressions of the candidate circulating miRNAs, especially at 2 days post immunization (dpi), 14 dpi, and 28 dpi. In addition, stress-induced immunosuppression significantly affected the immune response to NDV vaccine, which was manifested by significant changes in candidate circulating miRNAs at 2 dpi, 5 dpi, and 21 dpi. The featured expressions of candidate circulating miRNAs indicated their potential application as biomarkers in immunity and immunosuppression. Bioinformatics analysis revealed that the candidate circulating miRNAs possibly regulated immune function through key targeted genes, such as Mg^2+^/Mn^2+^-dependent 1A (*PPM1A*) and Nemo-like kinase (*NLK*), in the MAPK signaling pathway. This study provides a theoretical reference for studying the function and mechanism of circulating miRNAs in immune regulation.

## 1. Introduction

Stress has a negative impact on poultry as a result of its associated health problems, and the poultry industry is often plagued with stress-induced immunosuppression. Common stress factors are transportation, crowding, unsuited temperature, beak and toe breakage, and ammonia [1,2]. Studies have shown that microRNAs (miRNAs) are involved in the regulation of immunosuppression in the chicken thymus [3] and bursa of Fabricius [4], Large White pig lung [5], and rat heart [6]. Chickens are known to be frequently affected by stressors and immunosuppressive agents that impair organisms’ immunity in poultry industry [7]. However, the effect and mechanism of stress-induced immunosuppression affecting the immune response to vaccines have not been reported yet.

Newcastle disease (ND), caused by Newcastle disease virus (NDV), is one of the devastating diseases of poultry. A growing body of literature suggests that multiple miRNAs participate in the regulation of NDV infection [8,9]. For example, miR-203a and miR-451 target transglutaminase 2 (*TGM2*) and tyrosine 3-monooxygenase/tryptophan 5-monooxygenase activation protein zeta (*YWHAZ*), respectively, to reduce the expressions of inflammatory cytokines, thereby enhancing NDV replication [9,10]. On the other hand, miR-455-5p can inhibit suppressor of cytokine signaling 3 (*SOCS3*) to enhance the expressions of type I interferon and the interferon-inducible genes (*ISGs*), consequently suppressing NDV replication [11]. Furthermore, miR-375 targets ELAV-like RNA-binding protein 4 (*ELAVL4*) to regulate cell-cycle progression and reduce NDV proliferation [12]. Moreover, the structural protein genes of NDV have been found to be regulated by miRNAs (such as miR-375, miR-1603, and miR-1794) [12,13].

In this study, chickens were used as model animals to investigate the effect of stress-induced immunosuppression affecting the immune response to the NDV vaccine via circulating miRNAs. A dexamethasone (Dex)-simulated stress-induced immunosuppressed model and NDV vaccine-induced immune model were constructed, and the expression changes of 15 stress-induced immunosuppression [4,14,15] and NDV-related miRNAs (miR-124a-3p [9], miR-31-5p [9], miR-126-5p [9], miR-200b-3p [9], miR-122-5p [9], miR-30b-5p [9], miR-20a-5p [9], miR-34a-5p [9], miR-22-3p [9], miR-451 [10], miR-199-5p [10], miR-29b-3p [10], miR-19b-3p [10,16], miR-375 [12], and miR-198 [17]) were dynamically monitored in different treatment groups. The possible target genes and mechanisms of candidate miRNAs were then predicted. These results help to better understand the mechanism via which stress-induced immunosuppression affects the immune response to the NDV vaccine, and they provide a theoretical reference for further studying the function and mechanism of circulating miRNAs in immune regulation.

## 2. Materials and Methods

### 2.1. Experimental Grouping and Tissues Collection

A total of 160 1 day old nonimmunized Hy-Line Brown chickens were obtained from the Xiangfang hatchery in Harbin city, and equally divided into four groups: control group, Dex group, ND group, and Dex + ND group. A commercial diet and water were offered ad libitum, and different treatment groups were reared in isolation. From 7 to 11 days of age, chickens of the Dex group and Dex + ND group drank Dex every day (1.5 mg/kg). At 12 days old, chickens from the ND group and Dex + ND group were vaccinated with the NDV LaSota vaccine strain (Harbin Veterinary Research Institute, Harbin, China) via eye drop, and the control group and Dex group received the salt solution used to dilute the freeze-dried vaccine (Harbin Veterinary Research Institute, Harbin, China). At 1, 2, 3, 4, 5, 7, 14, 21, 28, and 35 days post immunization (dpi), the thymus, bursa of Fabricius, spleen, and blood samples were collected from three randomly selected chickens of each group. The blood was placed at a 45° tilt and 4 °C overnight in sterile round-bottom centrifuge tubes, and then centrifuged at 4 °C, 3000 rpm for 15 min to obtain serum. Other samples were frozen in liquid nitrogen and stored at −80 °C for subsequent analysis.

### 2.2. Antibody Level Determination and Organ Coefficient Analysis

Hemagglutination (HA) and hemagglutination inhibition (HI) assays were used to determine antibody levels of chicken serum with standard antigen of NDV LaSota strain (Harbin Veterinary Research Institute, Harbin, China) according to the method of Lukas Kaufmann et al. [18], and antibody titers were expressed as a reciprocal log_2_ value. The body weight, thymus, bursa of Fabricius, and spleen of three chickens were weighed, and organ coefficients were calculated according to the following formula: tissue weight (g)/body weight (g) × 100%.

### 2.3. Reverse Transcription and Quantitative Real-Time PCR (qRT-PCR)

Total RNA was extracted from different tissues using TRIzol (Invitrogen, Carlsbad, CA, USA) following the manufacturer’s instructions. Agarose gel electrophoresis (AGE) was used to estimate the integrity of total RNA, and a DU800 spectrophotometer (Beckman Coulter, Miami, FL, USA) was used to quantify the total RNA. Briefly, 300 ng of RNA of each sample was reverse-transcribed using the reverse transcription kit FSQ-301 (TOYOBO, Shanghai, China) following the manufacturer’s instructions. qRT-PCR amplification efficiency was assessed by varying the cDNA template concentration to obtain a relative quantitative standard curve, and specificity was assessed by melting curve. qRT-PCR was performed in three technical iterations with a 10 μL reaction mixture, which contained 5 μL of 2× SYBR Green I (TOYOBO, Shanghai, China), 0.2 μL of 50× ROX reference dye (TOYOBO, Shanghai, China), 0.3 μL of each primer, 1 μL of cDNA, and 3.2 μL of RNA-free water. *U6* was chosen as an endogenous control for miRNAs. The qRT-PCR procedure was as follows: 95 °C for 1 min, followed by 40 cycles at 95 °C for 15 s, 60 °C for 30 s, 72 °C for 30 s, and s final extension for 30 s at 72 °C. All primer sequences are provided in Table 1.

### 2.4. Bioinformatics Analysis

Only potential targeted genes that were predicted by TargetScan website v8.0 (www.targetscan.org, accessed on 14 July 2022) for each miRNA were further considered. These predicted target genes were subjected to enrichment analysis. TopGO was used for Gene Ontology (GO) annotation, and clusterProfiler R package v3.4.4 was used for Kyoto Encyclopedia of Genes and Genomes (KEGG) pathway analysis. Graphs were generated using the ggplot package in R.

### 2.5. Statistical Analysis

The 2^−∆∆Ct^ method was used to calculate the relative expression changes of the candidate miRNAs. The statistical significance was analyzed using an independent-sample *t*-test and one-way ANOVA in SPSS software v20.0 (IBM Inc., Armonk, NY, USA) after using Shapiro-wilk (SW) test to check the normality of distribution for each group of data samples. Charts were plotted using GraphPad Prism software (San Diego, CA, USA). In all analyses, *p* < 0.05 was taken to indicate statistical significance. 

## 3. Results

### 3.1. Analysis of Organ Coefficient and Serum Antibody

**Figure 1:**
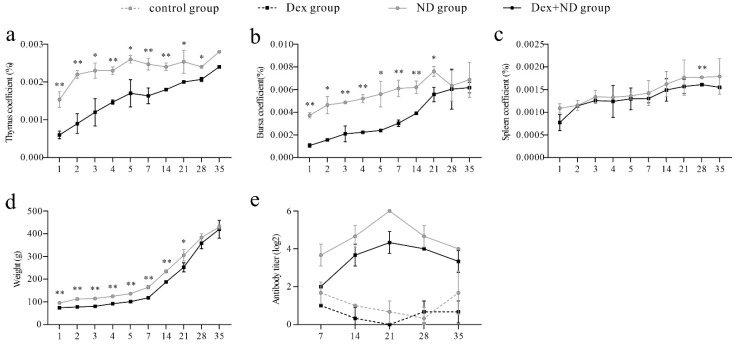
The identification results of immunosuppressed chicken model. (**a**–**d**) Comparison results of body weight and organ coefficient between ND group and Dex + ND group, respectively. * Significant difference between the two groups (*p* < 0.05); ** very significant difference between the two groups (*p* < 0.01). (**e**) Serum Newcastle disease virus (NDV) antibody titers in different treatment groups.

The results of the organ coefficients showed that the body weight, thymus, spleen, and bursa of Fabricius in the Dex + ND group were significantly lower than those in the ND group (*p* < 0.05) (Figure 1a–d). HI results showed that antibody titers in the ND group and Dex + ND group started to increase from 7 dpi onward, peaked on 21 dpi, and then decreased. Throughout the whole process, the antibody titers in the ND group were always higher than those in the Dex + ND group, while no NDV antibody was detected in the Dex group and control group (Figure 1e).

### 3.2. Differential Expression Analysis of Serum Circulating miRNAs between Dex Group and Control Group

**Figure 2:**
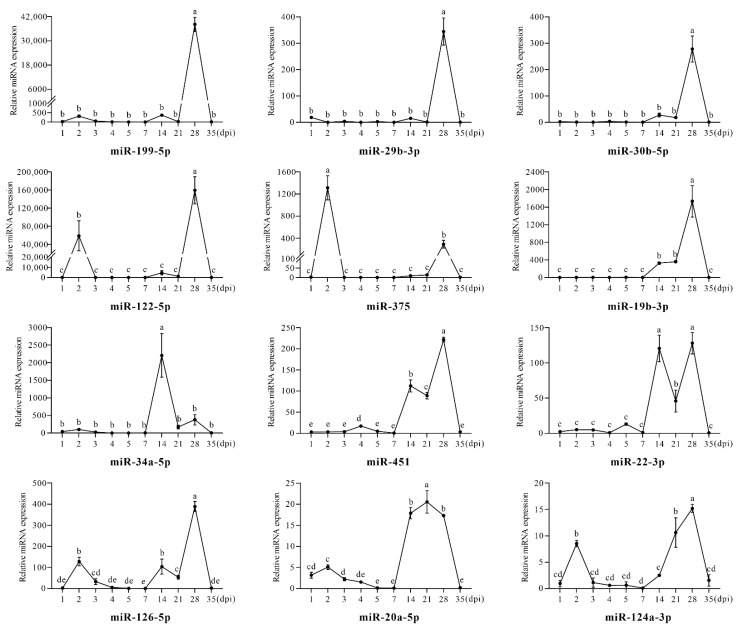
The relative expression levels of serum circulating miRNAs in Dex group. The different letters represent significant differences (*p* < 0.05).

The qRT-PCR results showed that serum circulating miR-198, miR-31-5p, and miR-200b-3p were not detected in either group; hence, follow-up analysis for the three miRNAs was not performed. The expression levels of the 12 remaining miRNAs were compared between the Dex group and control group, and the results showed that these miRNAs had similar trends at some timepoints. For example, at 2 dpi, miR-122-5p, miR-375, miR-126-5p, miR-20a-5p, and miR-124a-3p were significantly upregulated (*p* < 0.05); at 14 dpi, miR-19b-3p, miR-34a-5p, miR-451, miR-22-3p, miR-126-5p, miR-20a-5p, and miR-124a-3p were significantly upregulated (*p* < 0.05); at 28 dpi, all miRNAs except miR-20a-5p had significant upregulation trends (*p* < 0.05), with miR-199-5p, miR-29b-3p, and miR-122-5p upregulated more than 100-fold (Figure 2). Overall, 2 dpi, 14 dpi, and 28 dpi were the key timepoints for all candidate serum circulating miRNAs with the most significant upregulated changes.

It is worth mentioning that the changes of some miRNAs showed similar regularity. In the early stage of the Dex group (1–5 dpi), miR-199-5p, miR-29b-3p, miR-30b-5p, miR-19b-3p, miR-34a-5p, and miR-22-3p were not changed significantly, while miR-122-5p, miR-375, miR-126-5p, miR-20a-5p, and miR-124a-3p were significantly upregulated at 2 dpi, and only miR-451 was upregulated at 4 dpi. In the later stage of Dex treatment (7–35 dpi), miR-199-5p, miR-29b-3p, miR-30b-5p, miR-122-5p, and miR-375 were only significantly upregulated at 28 dpi, and miR-34a-5p was only significantly upregulated at 14 dpi. In addition, miR-19b-3p, miR-451, miR-22-3p, and miR-126-5p had similar trends, which were upregulated at 14 dpi and 28 dpi, respectively. On the other hand, miR-20a-5p and miR-124a-3p were continuously upregulated from 14 dpi to 28 dpi, showing upward trends.

### 3.3. The Differential Regulation of Circulating miRNAs in the Process of Stress-Induced Immunosuppression Affecting Immune Response to NDV Vaccine

**Figure 3:**
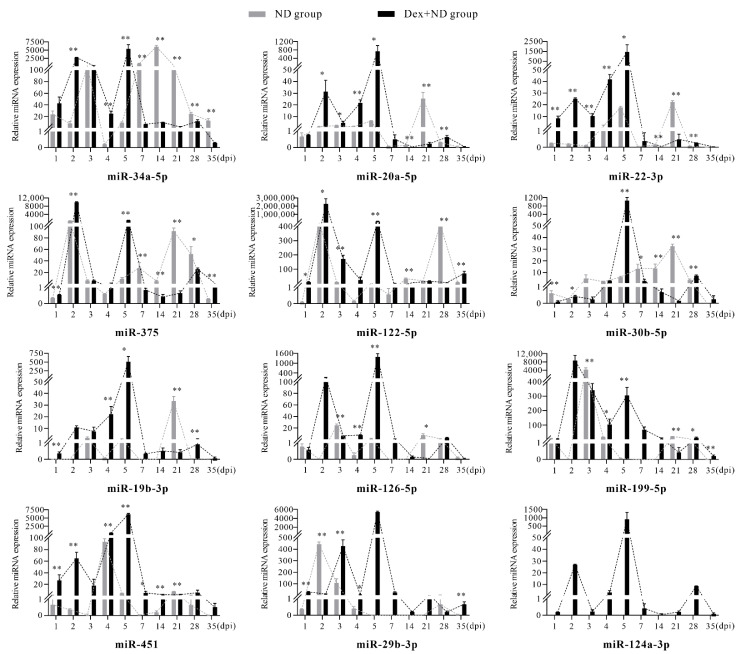
Comparison of the relative expression levels of serum circulating miRNAs between ND group and Dex + ND group. * Significant difference between the two groups (*p* < 0.05); ** very significant difference between the two groups (*p* < 0.01).

It must be noted that the expressions of miR-19b-3p, miR-126-5p, and miR-199-5p at 2 dpi, 7 dpi, and 14 dpi, miR-29b-3p at 5 dpi, 7 dpi, 14 dpi, and 21 dpi, and miR-124a-3p at all timepoints were not detected in the ND group; therefore, it was not possible to calculate normalized results for them at these timepoints. Through comparing the normalized expression results between the ND group and Dex + ND group, we found that the candidate circulating miRNAs showed a high degree of consistency at some timepoints (Figure 3).

In the phase of innate immunity (1–3 dpi) [19,20], qRT-PCR results showed that, compared with the ND group, miR-375, miR-122-5p, miR-30b-5p, miR-20a-5p, miR-451, miR-34a-5p, and miR-22-3p were significantly upregulated in the Dex + ND group (*p* < 0.05), among which miR-375 and miR-122-5p had the highest expression levels at 2 dpi. In addition, the expression levels of all candidate miRNAs, except for miR-29b-3p, were upregulated significantly at 2 dpi in the Dex + ND group, showing similar trends of upregulation (1–2 dpi) and downregulation (2–3 dpi). Overall, Dex had an inductive effect on expression levels of these candidate circulating miRNAs in the innate immune stage of the ND group, especially at 2 dpi.

In the transitional phase from innate immunity to acquired immunity (4–5 dpi), qRT-PCR results showed that, compared with ND group, all miRNAs were significantly upregulated in the Dex + ND group at 4 dpi and 5 dpi (*p* < 0.05). It is worth mentioning that miR-34a-5p, miR-20a-5p, miR-22-3p, miR-30b-5p, miR-19b-3p, miR-126-5p, miR-451, miR-29b-3p, and miR-124a-3p reached their expression peaks at 5 dpi, while miR-375, miR-122-5p, and miR-199-5p also showed obvious high expression. This indicated that the expression levels of candidate circulating miRNAs increased in the transition stage from innate immunity to acquired immunity of the ND group under Dex-induced immunosuppression. In addition, compared with 4 dpi, 5 dpi was a key timepoint to better reflect these changes.

In the acquired immune phase (7–35 dpi) [19,20], qRT-PCR results showed that, compared with the ND group, almost all candidate miRNAs were significantly downregulated in the Dex + ND group at 14 dpi and 21 dpi, while only miR-451 was upregulated at 14 dpi (*p* < 0.05). Furthermore, the timepoints with significant changes in circulating miRNAs were significantly different between the two groups. In the ND group, most circulating miRNAs had higher expression levels at 21 dpi, such as miR-20a-5p, miR-22-3p, miR-375, miR-30b-5p, miR-19b-3p, miR-126-5p, miR-199-5p, and miR-451, among which miR-20a-5p, miR-22-3p, miR-375, miR-30b-5p, and miR-19b-3p had the highest expression peaks in the whole process. In the Dex + ND group, the high expressions at 21 dpi were suppressed, and 28 dpi became the timepoint with relatively higher expression levels, as seen for miR-34a-5p, miR-20a-5p, miR-375, miR-30b-5p, miR-19b-3p, miR-126-5p, miR-451, and miR-124a-3p. Overall, Dex-induced immunosuppression downregulated the expression levels of candidate circulating miRNAs in acquired immunity of the Dex + ND group, and 21 dpi better reflected this repressive effect.

### 3.4. Functional Analysis and Pathway Prediction of Candidate Circulating miRNAs

**Figure 4:**
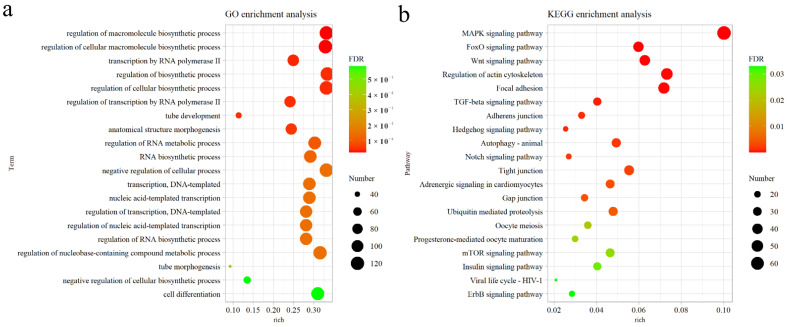
Functional enrichment analysis of genes targeted by 12 candidate miRNAs. (**a**) The top 20 enriched GO terms in BP (*p* < 0.05). (**b**) The top 20 enriched KEGG pathways (*p* < 0.05).

A total of 2233 genes targeted by the 12 candidate miRNAs (miR-34a-5p, miR-20a-5p, miR-22-3p, miR-375, miR-122-5p, miR-30b-5p, miR-19b-3p, miR-126-5p, miR-199-5p, miR-451, miR-29b-3p, and miR-124a-3p) were predicted using Targetscan software, and GO and KEGG analyses were performed on the basis of these genes (*p* < 0.05). GO analysis showed that the top 20 significantly enriched GO terms were mainly involved in the biological process (BP) of regulation of transcription by RNA polymerase II (Figure 4a). KEGG analysis found that these genes were enriched in multiple immune-related signaling pathways, including the MAPK signaling pathway, FoxO signaling pathway, Wnt signaling pathway, TGF-beta signaling pathway, Notch signaling pathway, mTOR signaling pathway, and ErbB signaling pathway. Among them, the MAPK signaling pathway was the most significant (Figure 4b). Therefore, the targeting relationship prediction between the candidate miRNAs and 67 predicted target genes engaged in the MAPK pathway indicated that protein phosphatase-related genes were the main targets; among them, the largest number of candidate miRNAs could target Mg^2+^/Mn^2+^-dependent 1A (*PPM1A*) and Nemo-like kinase (*NLK*). For example, miR-30b-5p, miR-20a-5p, miR-126-5p, miR-34a-5p, and miR-124a-3p could target *PPM1A*, while miR-375, miR-30b-5p, miR-126-5p, and miR-199-5p could target *NLK*.

## 4. Discussion

Intensive modern production models have resulted in animals being exposed to various forms of stresses, and stress can cause the body to produce glucocorticoids, which in turn lead to immunosuppression [21,22,23]. It has been demonstrated that Dex can be used as an immunosuppressant to explore the negative effects of high concentrations of glucocorticoids; therefore, using Dex to simulate stress-induced immunosuppression in poultry is reliable [24]. In this study, the change trends of body weight, organ coefficients, and antibody titers proved that the NDV immunity model and Dex-induced immunosuppressed model were successfully established. Interestingly, miR-198, miR-31-5p, and miR-200b-3p were not detected in the serum of either group; however, the expression changes of the three miRNAs after NDV infection was previously demonstrated [9,17]. The reason for their absence deserves further investigation. Hence, only the remaining 12 NDV-related miRNAs were used for follow-up studies.

To explore the dynamic changes of candidate circulating miRNAs after Dex treatment, we compared the expression levels of miRNAs between the Dex group and control group, and the results showed that 2 dpi, 14 dpi, and 28 dpi were the key timepoints with circulating miRNAs upregulated significantly, indicating that Dex had a stimulating effect on the expression levels of candidate miRNAs in serum. Furthermore, the effect of Dex-induced immunosuppression affecting the NDV immune response can be drawn from the expression changes of serum circulating miRNAs. In detail, almost all candidate miRNAs had similar expression trends in the Dex + ND group; 2 dpi, 4 dpi, and 5 dpi showed significant upregulation, while 14 dpi and 21 dpi showed significant downregulation. Among them, 2 dpi, 5 dpi, and 21 dpi were the most critical timepoints for Dex-induced immunosuppression affecting the NDV immune response because of their large change range. In addition, in a normal NDV immune response, 21 dpi in the acquired immune phase represented the relative expression peak of candidate circulating miRNAs. However, after Dex treatment, the expression levels of circulating miRNAs in the Dex + ND group were suppressed at this timepoint, and 2 dpi and 5 dpi became new expression peaks, providing new temporal clues for the detection of NDV vaccine efficacy in the immunosuppressed state.

Both in the innate immunity phase and in the transitional phase from innate immunity to acquired immunity, the expression levels of circulating miRNAs were significantly upregulated under Dex-induced immunosuppressive conditions. Studies have shown that miRNAs act as molecular switches in immune cells. MiR-122-5p inhibits the expression of activation-associated receptors in natural killer (NK) cells [25], and it also affects their degranulation activity [26]. Both miR-22-3p and miR-451 targeting tyrosine 3-monooxygenase/tryptophan 5-monooxygenase activation protein zeta (*YWHAZ*) cause dendritic cell (DC) apoptosis [27] and attenuate the secretion of proinflammatory cytokines, such as tumor necrosis factor (TNF) and interleukin 6 (IL-6) [28]. Moreover, miR-22-3p also targets histone deacetylase 4 (*HDAC4*) and p38 kinase (*p38*) to regulate DC activation [29] and antitumor ability [30]. On the other hand, miR-20a-5p can inhibit macrophage activation by targeting transforming growth factor beta receptor 2 (*TGFBR2*) [31] or inhibit mycobacteria-infected macrophages apoptosis by targeting mitogen-activated protein kinase 9 (*JNK2*) [32]. In addition, miRNAs are also able to control immune responses. For example, the miR-122-5p/TGF-beta activated kinase 1 (TAK1) axis negatively regulates the nuclear factor kappa B (NF-κB) and interferon regulatory factor 3 (IRF3) signaling pathways, thereby suppressing antiviral immune responses [33]; miR-375 is a key miRNA in response to stress-induced immunosuppression [4,15]. Therefore, we speculated that stress-induced immunosuppression affected the NDV innate immune response by upregulating the expression levels of these circulating miRNAs.

In the phase of acquired immunity, Dex led to a significant downregulation of candidate circulating miRNA expression. Studies have shown that a variety of miRNAs play roles in the regulation of immune cells, including proliferation, activation, and differentiation. For example, miR-20a-5p alters T-cell proliferation by targeting mitogen-activated protein kinase 1 (MAPK1) [34], and miR-375 induces DC maturation through the JAK2–STAT3 signaling pathway, indirectly increasing CD4^+^ T cells and CD8^+^ T cells [35]; miR-34a-5p and miR-22-3p downregulate the expressions of programmed cell death 1 ligand 1 (*PD-L1*) and phosphatase and tensin homolog (*PTEN*) in T cells and B cells, thereby improving the reactivities of T cells [36] and B cells [37], respectively. Furthermore, miR-22-3p as a signature of the maturation of T follicular helper (Tfh) cells, is upregulated during the differentiation of Tfh cells [38], while miR-126-5p enhances the Notch1 signaling pathway-mediated differentiation of CD4^+^ T cells by targeting delta-like noncanonical Notch ligand 1 (*DLK1*) [39]. Thus, we speculated that stress-induced immunosuppression affected NDV acquired immune response by downregulating the expression levels of these circulating miRNAs, thus suppressing the immune response. Interestingly, miR-20a-5p, miR-22-3p, miR-375, and miR-34a-5p emerged as the best candidates for biomarkers, because they had highly similar significant changes and a strong association with immunity.

Bioinformatics analysis showed that multiple candidate miRNAs were predicted to target *PPM1A* and *NLK* genes. PPM1A, a member of intracellular serine/threonine protein phosphatases, is an important regulator of host immunity in response to pathogens [40,41]. PPM1A was identified as the first known phosphatase of mitochondrial antiviral signaling protein (MAVS), which can silence cytosolic RNA sensing and antiviral defense through direct dephosphorylation of MAVS and TANK-binding kinase 1 (TBK1) [42], while also inhibiting the NF-κB pathway [43], leading to virus escape from host immune surveillance of viral replication. NLK, an evolutionarily conserved serine/threonine mitogen-activated protein kinase (MAPK), is established as an important regulator in diverse cellular processes [44]. Studies have shown that NLK suppresses antiviral immune responses by phosphorylating MAVS, leading to its degradation [45,46], in addition to negatively regulating the NF-κB pathway [47]. In short, PPM1A and NLK are essential regulators in stress response pathways. Therefore, we speculated that our candidate miRNAs can potentially target *PPM1A* and *NLK* to regulate immune function through the NF-κB and MAPK signaling pathways, representing an important mechanism of stress-induced immunosuppression negatively regulating the NDV adaptive immune response.

## 5. Conclusions

In conclusion, Dex-induced immunosuppression can significantly affect the expression levels of candidate circulating miRNAs in the NDV vaccine immune response, which were upregulated at 2 dpi and 5 dpi but downregulated at 21 dpi. Our data suggest that the possible mechanism of stress-induced immunosuppression affecting the immune response to the NDV vaccine is the potential targeting of *NLK* and *PPM1A* in the MAPK signaling pathway by candidate miRNAs. This study lays the foundation for an in-depth study of the function and mechanism of circulating miRNAs in vaccine immune response under stress-induced immunosuppression.

## Figures and Tables

**Table 1:** Primers used to detect microRNAs (miRNAs) expression levels with qRT-PCR.

miRNA	Primer (5′–3′)
miR-375	RT-CTCAACTGGTGTCGTGGAGTCGGCAATTCAGTTGAGTAACGCGA
	F-ACACTCCAGCTGGGTTTGTTCGTTCGGCTC
miR-20a-5p	RT-CTCAACTGGTGTCGTGGAGTCGGCAATTCAGTTGAGCTACCTGC
	F-ACACTCCAGCTGGGTAAAGTGCTTATAGTGC
miR-451	RT-CTCAACTGGTGTCGTGGAGTCGGCAATTCAGTTGAGAAACTCAG
	F-ACACTCCAGCTGGGAAACCGTTACCATTACT
miR-124a-3p	RT-CTCAACTGGTGTCGTGGAGTCGGCAATTCAGTTGAGTGGCATTC
	F-ACACTCCAGCTGGGTTAAGGCACGCGGTGA
miR-199-5p	RT-CTCAACTGGTGTCGTGGAGTCGGCAATTCAGTTGAGGAACAGGT
	F-ACACTCCAGCTGGGCCCAGTGTTCAGACTAT
miR-126-5p	RT-CTCAACTGGTGTCGTGGAGTCGGCAATTCAGTTGAGCGCGTACC
	F-ACACTCCAGCTGGGCATTATTACTTTTGG
miR-19b-3p	RT-CTCAACTGGTGTCGTGGAGTCGGCAATTCAGTTGAGTCAGTTTT
	F-ACACTCCAGCTGGGTGTGCAAATCCATGCAA
miR-200b-3p	RT-CTCAACTGGTGTCGTGGAGTCGGCAATTCAGTTGAGATCATCAT
	F-ACACTCCAGCTGGGTAATACTGCCTGGTAAT
miR-34a-5p	RT-CTCAACTGGTGTCGTGGAGTCGGCAATTCAGTTGAGAACAACCA
	F-ACACTCCAGCTGGGTGGCAGTGTCTTAGCTG
miR-122-5p	RT-CTCAACTGGTGTCGTGGAGTCGGCAATTCAGTTGAGACAAACAC
	F-ACACTCCAGCTGGGTGGAGTGTGACAATGGT
miR-22-3p	RT-CTCAACTGGTGTCGTGGAGTCGGCAATTCAGTTGAGACAGTTCT
	F-ACACTCCAGCTGGGAAGCTGCCAGTTGAAG
miR-31-5p	RT-CTCAACTGGTGTCGTGGAGTCGGCAATTCAGTTGAGCAGCTATG
	F-ACACTCCAGCTGGGAGGCAAGATGTTGGCA
miR-30b-5p	RT-CTCAACTGGTGTCGTGGAGTCGGCAATTCAGTTGAGAGCTGAGT
	F-ACACTCCAGCTGGGTGTAAACATCCTACAC
miR-198	RT-CTCAACTGGTGTCGTGGAGTCGGCAATTCAGTTGAGGAACCTAT
	F-ACACTCCAGCTGGGGGTCCAGAGGGGAGAT
miR-29b-3p	RT-CTCAACTGGTGTCGTGGAGTCGGCAATTCAGTTGAGAACACTGA
	F-ACACTCCAGCTGGGTAGCACCATTTGAAATC
*U6*	F-CTCGCTTCGGCAGCACA
	R-AACGCTTCACGAATTTGCGT
miRNA	R-TGGTGTCGTGGAGTCG
